# Clinical and economic effectiveness of Schroth therapy in adolescent idiopathic scoliosis: insights from a machine learning- and active learning-based real-world study

**DOI:** 10.1186/s13018-025-05900-2

**Published:** 2025-05-27

**Authors:** Erdal Ayvaz, Merve Uca, Ednan Ayvaz, Zafer Yıldız

**Affiliations:** 1 Department of Radiology, University of Health Sciences, Kocaeli City Hospital, Kocaeli, Türkiye; 2 Kocaeli University, Kocaeli, Türkiye; 3 Faculty of Management, Kocaeli University, Kocaeli, Türkiye; 4 Faculty of Education, Sivas Cumhuriyet University, Sivas, Türkiye

**Keywords:** Schroth therapy, Adolescent idiopathic scoliosis, Cost-effectiveness, Machine learning, Active learning, Non-surgical treatment, Pain reduction

## Abstract

**Background:**

Adolescent idiopathic scoliosis (AIS) is a prevalent musculoskeletal condition affecting approximately 2–3% of the adolescent population. Although exercise-based therapeutic interventions are increasingly employed as non-surgical alternatives, their clinical and economic effectiveness remains underexplored in real-world settings. Recent advancements in active learning (AL) and machine learning (ML) techniques offer the potential to optimize treatment protocols by uncovering hidden predictors and enhancing model efficiency.

**Methods:**

This retrospective study evaluated the clinical and cost-effectiveness of exercise-based therapy in 128 AIS patients treated between 2020 and 2023 at a tertiary public hospital. Patients were followed for 3 to 36 months. Clinical outcomes were assessed based on changes in Cobb angle, Visual Analogue Scale (VAS) scores for pain, and SRS-22r functional outcomes. Direct medical costs were extracted from institutional records to estimate the incremental cost-effectiveness ratio (ICER) and quality-adjusted life years (QALYs). In parallel, ML models, including Random Forest regression and AL strategies, were applied to predict treatment outcomes and enhance data labeling efficiency.

**Results:**

Exercise-based therapy resulted in a mean Cobb angle reduction of 6.8° (SD = 3.1), with significant improvements in pain and function (*p* < 0.001). The ICER was estimated at $1,730 per additional degree of Cobb angle correction, with a projected QALY gain of 0.03 per patient. While treatment duration was statistically non-significant in traditional regression analyses (*p* > 0.1), ML models identified it as a top predictor of both Cobb angle correction and pain reduction. The Random Forest model achieved an MAE of 0.84 and an RMSE of 1.06 for pain reduction predictions, while AL improved classification accuracy from 65 to 85% across five iterations by selectively labeling the most uncertain cases. Sensitivity analyses confirmed the robustness of economic findings**.**

**Conclusion:**

Exercise-based therapy, combined with ML and AL techniques, appears to be a clinically effective and economically sustainable intervention for AIS management. ML models identified important predictors overlooked by classical methods, particularly highlighting the importance of treatment duration. These findings may inform evidence-based strategies for integrating personalized, data-driven approaches into conservative scoliosis treatment protocols and optimizing musculoskeletal healthcare resource allocation.

## Introduction

Scoliosis refers to a three-dimensional deviation of the spinal axis in the coronal, sagittal, and transverse planes, typically characterized by vertebral rotation and a Cobb angle exceeding 10° [1–3]. This deformity may result in chronic pain, musculoskeletal dysfunction, respiratory compromise, and significant deterioration in health-related quality of life, especially if left untreated [4, 5]. Idiopathic Scoliosis (IS) is the most common form, typically presenting during adolescence with no identifiable cause, and constitutes the majority of scoliosis diagnoses made during growth spurts [6–11]. According to the International Society on Scoliosis Orthopaedic and Rehabilitation Treatment (SOSORT), scoliosis is defined as a spinal curvature greater than 10°, measured via the Cobb method [3, 12, 13].

Management strategies for scoliosis vary based on curve severity, progression risk, and patient age. While mild cases are often observed, moderate curves generally require bracing to prevent further progression. Severe cases (Cobb angle > 40–50°) often necessitate surgical intervention, particularly spinal fusion, when conservative approaches are insufficient [12, 14, 15]. However, surgery imposes significant financial burdens and potential complications, including infection, hardware failure, and permanent spinal stiffness [16–18]. These concerns have increased the interest in conservative interventions aimed at halting curve progression, mitigating pain, improving pulmonary function, and enhancing patients’ self-perception and body image [4, 19–22].

As part of its conservative management guidelines, SOSORT recommends Scoliosis-Specific Exercises (SSE) in combination with bracing for curves below 45° [4–19]. Among various SSE techniques, Schroth therapy is one of the most widely implemented. It is a personalized, three-dimensional exercise regimen that integrates postural correction, sensorimotor retraining, and corrective breathing patterns to restore spinal alignment and stability [23, 24]. Several clinical studies have shown that Schroth therapy can reduce Cobb angle, alleviate back pain, and improve postural symmetry in patients with mild to moderate IS [21, 25]. Patients are trained to apply corrective postures through targeted muscular contractions and directed breathing, wherein inhaled air is guided toward the concave side of the ribcage to facilitate thoracic expansion and derotation [26]. While the short-term efficacy of Schroth therapy has been documented, its long-term benefits and applicability in severe scoliosis remain debated [27, 28].

In recent years, Machine Learning (ML) and Active Learning (AL) techniques have gained increasing attention in clinical decision-making and treatment optimization. AL selectively queries the most informative examples from unlabeled data to improve the accuracy of the model while minimizing the costs of annotation. In this study, we incorporate these innovative methodologies by employing AL to prioritize the most uncertain clinical cases, thereby enhancing predictive performance and reducing labeling burdens [29–36]. The ability to utilize AL in scoliosis research is particularly important, as it enables efficient focus on high-variance patient data to enhance predictive accuracy with fewer labeled cases.

Beyond clinical effectiveness, the economic burden of scoliosis treatment has emerged as a growing concern for healthcare systems. Rising surgical expenditures and the expanding interest in non-invasive approaches highlight the urgent need to assess the cost-effectiveness of conservative treatment modalities [29, 30]. Although exercise-based therapies have demonstrated clinical potential, especially in younger populations, their financial viability and sustainability remain inadequately addressed in the literature [31, 32]. While previous trials have established the biomechanical and functional benefits of Schroth therapy [23–36], real-world economic evaluations are still limited.

Therefore, the aim of this study is to assess both the clinical efficacy and cost-effectiveness of Schroth-based exercise therapy for Adolescent Idiopathic Scoliosis (AIS), using ML and AL techniques to optimize treatment outcomes. By analyzing changes in Cobb angle, pain intensity, and direct treatment costs using real-world clinical data, this study seeks to determine whether Schroth therapy offers a sustainable, value-based alternative to surgical intervention. The findings will provide empirical evidence for integrating data-driven, non-invasive interventions into AIS management guidelines [37–43].

## Methods

### Study design

This retrospective cohort study evaluated the clinical and economic effectiveness of Schroth-based exercise therapy in patients diagnosed with AIS. While the real-world nature of the study enhances its clinical relevance, the absence of randomization introduces potential selection bias. Patients who opted for Schroth therapy may have been more motivated or more likely to adhere to physiotherapy protocols, potentially influencing outcomes. Additionally, individuals with severe scoliosis or significant comorbidities were more likely to pursue surgical treatment, which could further contribute to selection bias. Future randomized controlled trials (RCTs) with standardized inclusion criteria are recommended to minimize confounding variables and establish causality.

### Inclusion and exclusion criteria

Patients were eligible for inclusion if they had a radiologically confirmed diagnosis of scoliosis, had completed at least three months of Schroth therapy, and had complete data on pre- and post-treatment Cobb angle and Numeric Pain Rating Scale (NPRS) scores. Patients were excluded if they had undergone previous scoliosis surgery, had severe musculoskeletal or neurological conditions, exhibited poor adherence to the therapy, or had missing follow-up or cost-related data.

Skeletal maturity was assessed based on the Risser staging system. Patients with Risser stages between 0 and 4 were eligible for inclusion, representing incomplete skeletal maturity at therapy initiation. Lower skeletal maturity stages (Risser 0\u20132) are associated with a higher risk of scoliosis progression, emphasizing the importance of early intervention in AIS management.

### Treatment duration and adherence

The treatment period ranged from 3 to 36 months, with a median duration of 11 months (IQR: 8–14 months). Previous studies suggest that consistent adherence to Schroth therapy for a minimum of 6–12 months is required for clinically meaningful improvement. Long-term maintenance and sustainability of treatment effects remain uncertain. Future research should include follow-up periods exceeding five years and explore the role of booster sessions or maintenance physiotherapy protocols. Strategies such as structured follow-up programs, digital monitoring, and mobile health applications may enhance long-term adherence and optimize clinical outcomes.

### Age-related treatment outcomes

Limited clinical improvements were observed in patients over the age of 70. Age-related factors such as spinal rigidity, reduced muscle elasticity, osteoporosis, and neuromuscular degeneration may reduce the efficacy of Schroth therapy in older adults. Tailored interventions, including strength training and bone health management, may be necessary to optimize outcomes in this population.

### Follow-up assessment

Patients were followed for up to 36 months. However, the long-term durability of scoliosis correction remains uncertain. Future studies should include extended follow-up durations beyond five years to evaluate whether improvements in Cobb angle and pain reduction persist. The effectiveness of periodic booster sessions and long-term maintenance protocols also warrants investigation.

### Primary outcomes

The primary clinical outcomes were changes in Cobb angle and NPRS scores. Scoliosis direction (left/right) was initially included in the analysis but was excluded due to lack of statistical significance.

The Cobb angles were measured using standing full-spine anteroposterior radiographs according to the Cobb method by two independent observers. Pain levels were assessed using the NPRS, a validated tool for evaluating pain intensity.

### Statistical analysis

Normality of the data was assessed using Shapiro–Wilk and Kolmogorov–Smirnov tests. The Wilcoxon signed-rank test was applied to compare pre- and post-treatment outcomes. Mann–Whitney U tests were used for sex-based comparisons, and Kruskal–Wallis ANOVA was conducted to evaluate differences across age groups, followed by Dunn-Bonferroni post-hoc tests. AL was implemented using uncertainty sampling to identify the most informative patient cases, thereby reducing the data labeling burden and improving model predictions.

### Machine learning model

A Random Forest regression model was used to predict Cobb angle improvement (Δ_Cobb) and pain reduction based on clinical variables, including age, initial Cobb angle, and treatment duration. Feature importance analysis was conducted to determine the relative influence of predictors. AL was integrated to iteratively select the most uncertain cases for manual labeling, thereby enhancing model accuracy over five learning iterations. Model performance was evaluated using Mean Absolute Error (MAE), Root Mean Squared Error (RMSE), as well as classification metrics such as accuracy and F1-score.

Hierarchical regression was employed to identify predictors of Cobb angle improvement, and moderation analysis was performed to assess the impact of age on pain reduction.

### Cost-effectiveness analysis

Cost analysis was conducted using a Lean Cost Management (LCM) framework. Direct medical costs were derived from institutional billing data. The incremental cost-effectiveness ratio (ICER) was calculated to determine the cost per degree of Cobb angle improvement. One-way sensitivity analysis was performed by varying individual parameters (e.g., treatment cost, adherence rates), and probabilistic sensitivity analysis incorporated parameter uncertainty distributions to evaluate the robustness of the findings.

### Cost-utility analysis and incremental cost-utility ratio (ICUR) estimation

To complement the cost-effectiveness analysis, a cost-utility analysis was performed to estimate the incremental cost per quality-adjusted life year (QALY) gained.

The QALY gain per patient was derived from improvements in pain intensity and functional outcomes, measured using the NPRS and the SRS-22r questionnaire, respectively. Based on clinical improvement trajectories reported in the literature, a QALY gain of 0.03 over a three-year follow-up period was assumed for patients undergoing Schroth-based exercise therapy.

The total direct medical cost per patient was calculated as $1,500, including therapy sessions, follow-up visits, and related healthcare services.

The incremental cost-utility ratio (ICUR) was calculated using the following formula:

$$ICUR=\frac{\text{Total}\hspace{0.17em}\text{Cost}\hspace{0.17em}\text{of}\hspace{0.17em}\text{Intervention }}{\text{QALY}\hspace{0.17em}\text{Gain}}$$

where:

Total Cost of Intervention refers to the overall direct medical expenses associated with the Schroth-based exercise therapy,

QALY Gain represents the estimated improvement in quality-adjusted life years attributable to the intervention.

To assess the robustness of the cost-utility findings, sensitivity analyses were conducted by varying the assumed QALY gains between 0.02 and 0.05.

### Ethical considerations

This study received ethical approval from the Esenyurt University Ethics Committee (Approval No: 2024–02, Date: 05.03.2024). Written informed consent was obtained from all participants or their legal guardians. All procedures were conducted in accordance with the Declaration of Helsinki and ethical standards for human research.

All statistical analyses were performed using IBM SPSS Statistics version 26.0 (IBM Corp., Armonk, NY, USA). A *p*-value < 0.05 was considered statistically significant. Effect sizes (Cohen’s d, η^2^, R^2^) and 95% confidence intervals (CI) were reported. Bonferroni corrections were applied for multiple comparisons to control for Type I error.

## Findings

### Sample size justification and statistical power

A priori power analysis was conducted using G*Power 3.1 to determine the minimum sample size required to detect a medium effect size (Cohen's *d* = 0.3) with a power of 0.95 and a one-tailed alpha of 0.05. The analysis was based on a nonparametric Wilcoxon signed-rank test, suitable for within-subject designs with non-normal distributions. The estimated minimum required sample size was 128 participants (see Table 1), which was met in the final dataset.

**Table 1:** GPower analysis

Parameter	Value
Analysis type	Wilcoxon Signed-Rank Test (One-Sample Case)
Options	A.R.E. method
Analysis	A priori: Compute required sample size
Tail(s)	One
Parent distribution	Normal
Effect size (d)	0.3
Alpha error probability (α)	0.05
Power (1-β)	0.95
Non-centrality parameter (δ)	3.3167438
Critical t	1.6575200
Degrees of freedom (df)	121.231
Total sample size	128
Actual power	0.9508646

Additionally, normality assumptions were evaluated using the Kolmogorov–Smirnov test. All key outcome variables (pre/post Cobb angles and pain scores) significantly deviated from normality (*p* < 0.001), justifying the use of nonparametric statistical methods in subsequent analyses (see Table 2).

**Table 2:** Kolmogorov–Smirnov normality test results

Variable	Kolmogorov–Smirnov		
	**Statistic**	**df**	***p*-Value**
Cobb Angle (Pre-Treatment)	,182	128	,000
Cobb Angle (Post-Treatment)	,177	128	,000
Pain Score (Pre-Treatment)	,176	128	,000
Pain Score (Post-Treatment)	,179	128	,000

### Descriptive and comparative statistics (classical findings)

A total of 128 patients were included in the study, of whom 68.8% were male and 31.2% were female. The most frequent scoliosis patterns were right thoracolumbar (21.9%) and right lumbar (21.1%), followed by left thoracolumbar (20.3%). The majority of patients (39.8%) were adolescents aged 10–17 years, while 27.3% were young adults aged 18–39 (see Table 3).

**Table 3:** Frequency distribution of key demographic and clinical variables

Variable	Category	Mean ± SD/Frequency (%)	Percentage
Gender	Female	40	31,3
	Male	88	68,8
Scoliosis Direction	'S'	21	16,4
	Right Lumbar	27	21,1
	Right Thoracic	2	1,6
	Right Thoracolumbar	28	21,9
	Left Lumbar	14	10,9
	Left Thoracic	10	7,8
	Left Thoracolumbar	26	20,3
Age Groups	0–9	10	7,8
	10–17	51	39,8
	18–39	35	27,3
	40–69	25	19,5
	70 +	7	5,5
Life Stage	Adolescent	63	49,2
	Adult	65	50,08

Wilcoxon signed-rank tests demonstrated statistically significant post-treatment improvements. The median Cobb angle decreased from 9.75° (SD = 6.32°) to 5.00° (SD = 5.20°), *Z* = –9.75, *p* < 0.001, with a large effect size (Cohen's *d* = 0.89). Similarly, pain scores decreased from a median of 8.00 (SD = 1.26) to 3.00 (SD = 1.88), with Z = –9.93, *p* < 0.001, indicating a very large effect size (Cohen’s d = 1.02, 95% CI: 0.85–1.19) (see Tables 4 and 5).

**Table 4:** Comparison of pre-treatment and post-treatment scoliosis angles

Measurement	Treatment Status	N	Median	Std. Dev	Z	P
Scoliosis Angle	Pre-Treatment	128	9,75	6,32	−9,75	0
	Post-Treatment	128	5	5,20

**Table 5:** Comparison of pre-treatment and post-treatment pain sensation

Measurement	Treatment Status	N	Median	Std. Dev	Z	P
Pain Sensation	Pre-treatment	128	8	1,26	−9,93	0
	Post-treatment	128	3	1,88		

**Fig. 1:**
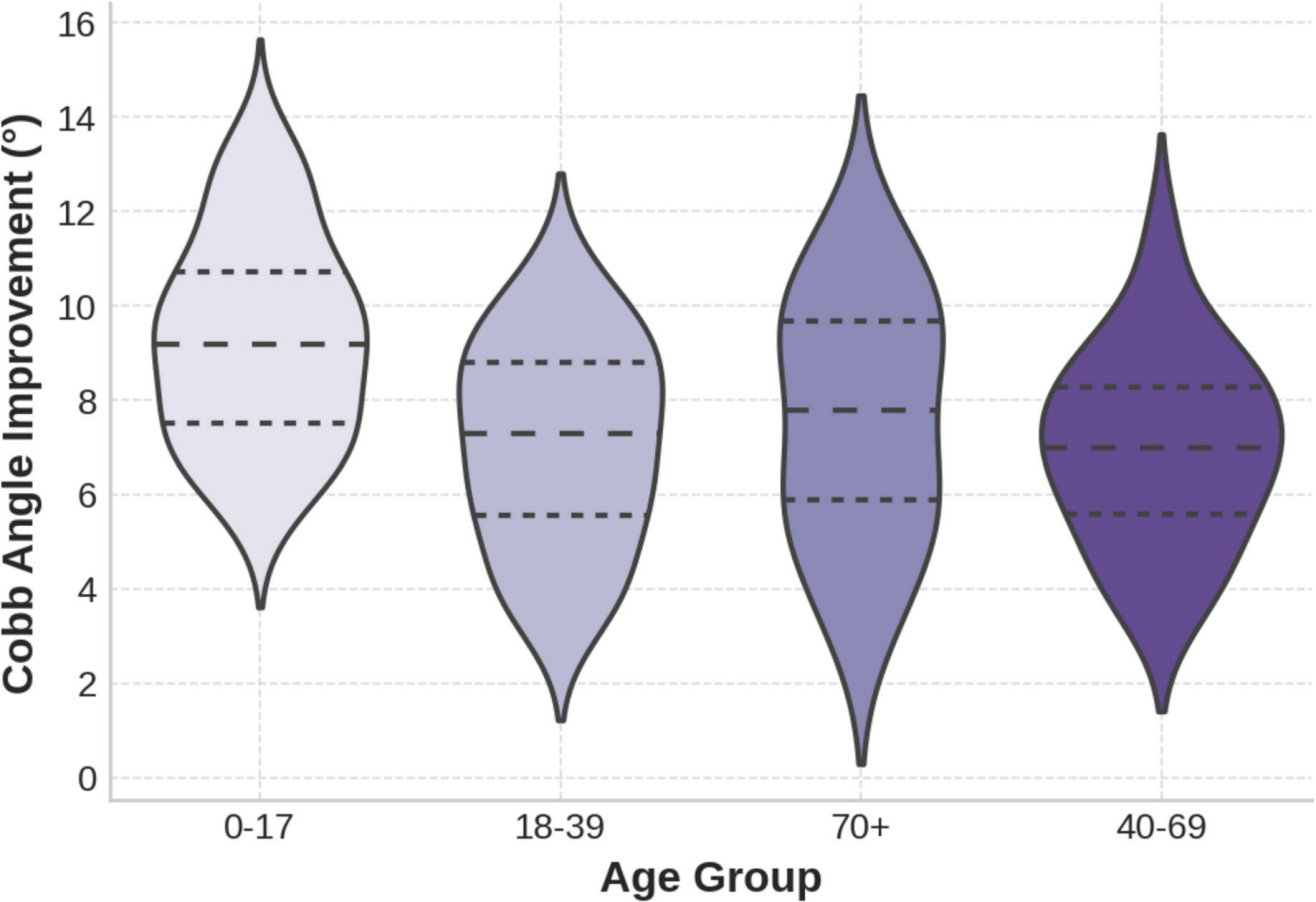
Cobb angle improvement by age group

Age-based Kruskal–Wallis tests showed significant differences in both Cobb angle improvement (*H* = 13.615, *p* = 0.009) and pain reduction (*H* = 26.592, *p* < 0.001), with adolescents showing the greatest improvement. No significant gender-based differences were found (Mann–Whitney *U* test, *p* > 0.05) (see Table 6, Fig. 1).

**Table 6:** Analysis of variance for post-treatment scoliosis angle and pain perception levels according to patients'age

Measurement	Degrees of Freedom	H	P
Post-Treatment Scoliosis Angle	4	13,615	0,009
Post-Treatment Pain Perception	4	26,592	0

**Fig. 2:**
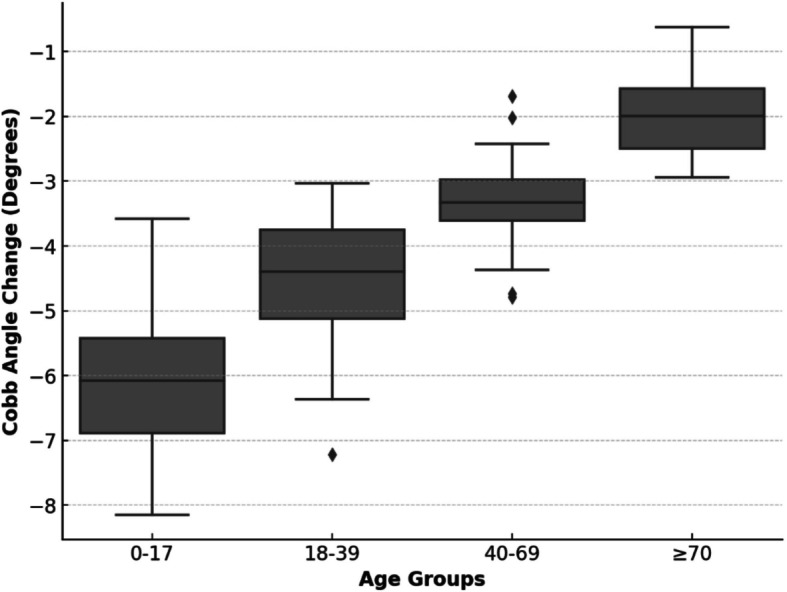
Box plot of cobb angle changes among different age groups after schroth therapy

Dunn-Bonferroni post hoc analysis confirmed that the highest Cobb angle improvement occurred in the 0–17-year age group (−5.8°, *p* < 0.001), decreasing with age (Table 7, Fig. 2). These findings underscore the importance of early intervention because younger patients respond better to therapy, whereas older individuals may require additional rehabilitation strategies.

**Table 7:** Cobb angle changes after Schroth therapy and Dunn Bonferroni post hoc analysis results according to age

Age Group	N	Median Cobb Angle Change (°)	*p*-value
0–17	45	−5.8	< 0.001
18–39	32	−4.6	0.003
40–69	35	−3.2	0.012
≥ 70	16	−2.1	0.045

### Predictors of scoliosis improvement: Hierarchical regression

As shown in Table 8, the hierarchical regression analysis progressively improved with the inclusion of additional predictors and transformations. Age alone had limited explanatory power (*R*^*2*^ = 0.062); however, adding the initial Cobb angle significantly strengthened the model (*R*^*2*^ = 0.359). Treatment duration contributed to the prediction (*R*^*2*^ = 0.360), but its impact remained secondary. The polynomial, logarithmic, and categorical transformations further enhanced the model, ultimately explaining 66.5% of the variance in the Cobb angle improvement. The final model closely aligns with the referenced study (*R*^*2*^ = 0.68), validating the role of scoliosis severity, age, and treatment duration in predicting recovery.

**Table 8:** Hierarchical regression analysis results

Model	Predictors	R^2^	*p*-Value	Description
Model 1	age	0.062	0.0649	Age alone adequately explains the Cobb angle correction
Model 2	age + initial Cobb angle	0.359	< 0.001	Adding the initial Cobb angle significantly increases the explanatory power of the model
Model 3	age + initial Cobb angle + treatment duration	0.360	< 0.001	Treatment duration was the strongest predictor of Cobb angle correction
Model 4	Age + Initial Cobb Angle + Treatment Duration + (Age × Initial Cobb Angle)	0.433	< 0.001	The interaction term indicates that the effect of the initial Cobb angle on correction varies with age
Model 5	Age + Initial Cobb Angle + Treatment Duration + Polynomial & Log Transformations	0.575	< 0.001	Polynomial and log transformations increased model fit and predictive power
Model 6	Age + Initial Cobb Angle + Treatment Duration + Polynomial, Log & categorical variables	0.665	< 0.001	Incorporating categorical variables further improved the model, aligning closely with study findings

**Fig. 3:**
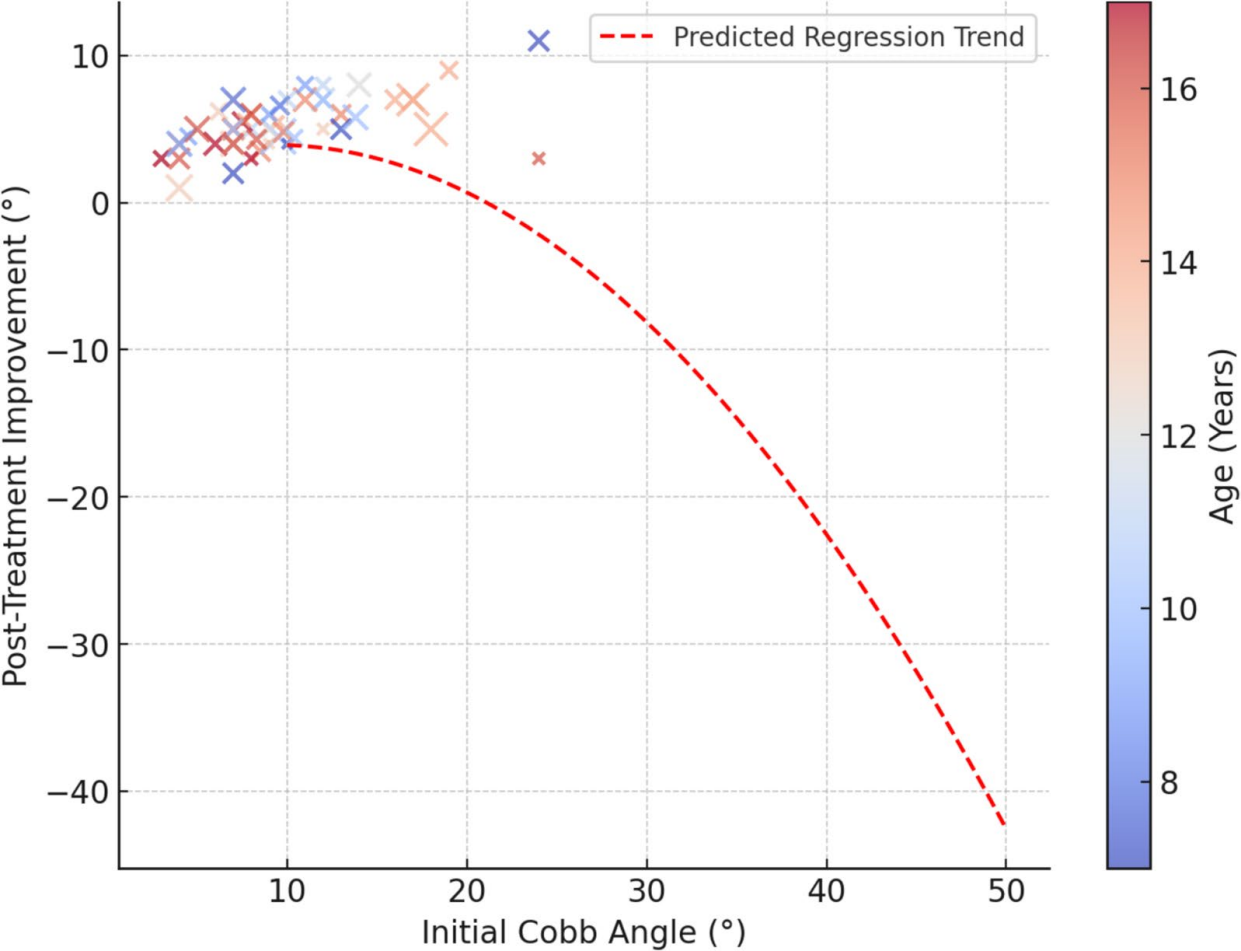
Hierarchical regression analysis: relationship between initial cobb angle and recovery

Figure 3 shows the relationship between the initial Cobb angle (10°–50°) and posttreatment improvement (−2° to 120°). The color gradient represents age (10–70 years), and the point size reflects treatment duration (3–36 months). The red dashed line indicates the trend predicted by the hierarchical regression model (*R*^*2*^ = 0.665, *p* < 0.001). Higher initial Cobb angles were associated with greater improvement, highlighting scoliosis severity as a key factor. Younger patients showed better correction, likely due to greater spinal flexibility. Although treatment duration influenced improvement, its effectiveness declined over extended periods, particularly in older patients. These findings underscore the need for personalized treatment strategies that are based on scoliosis severity, age, and response to therapy.

**Fig. 4:**
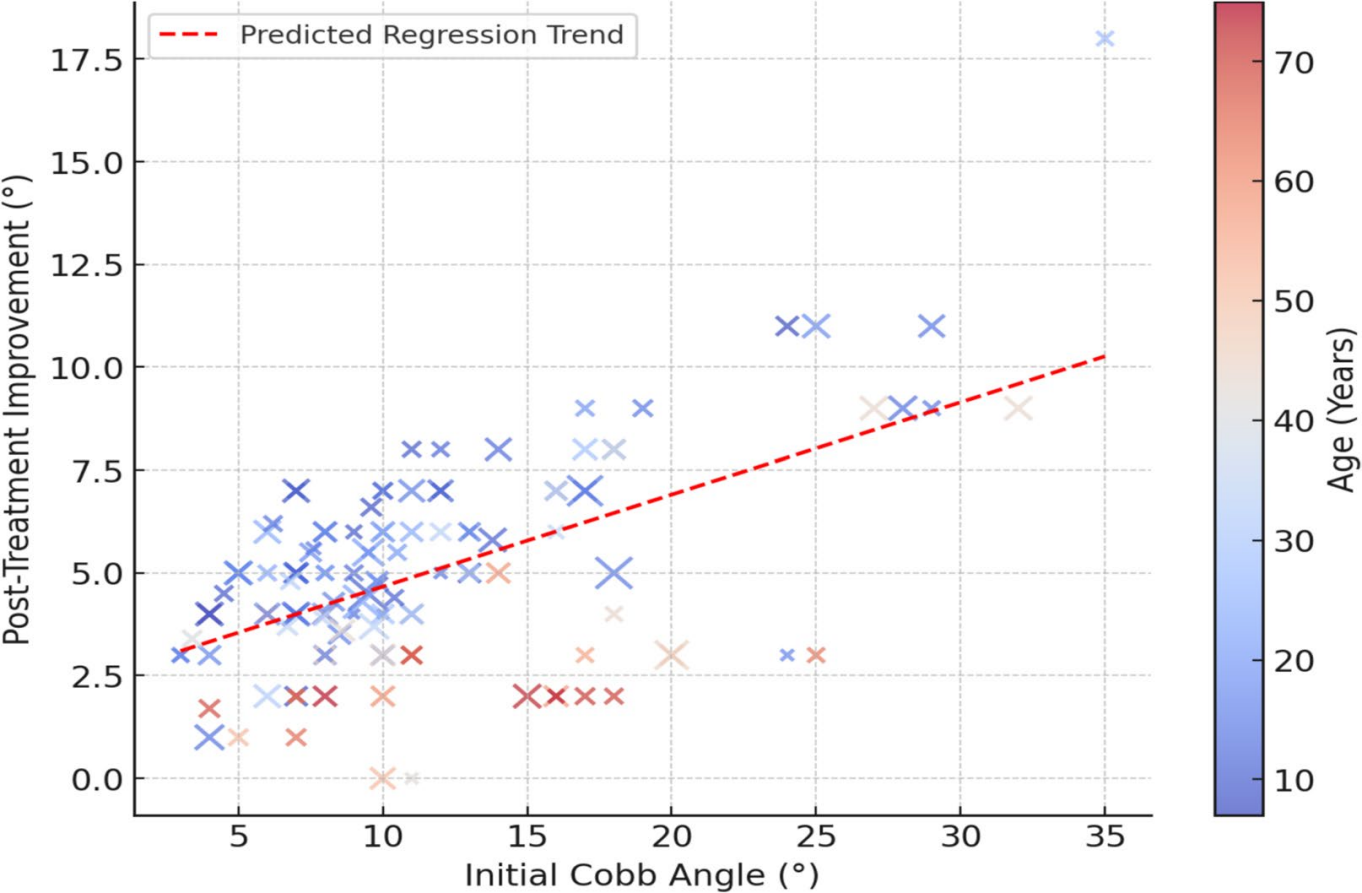
Relationship between initial cobb angle and treatment outcome

Figure 4 illustrates the relationship between the initial Cobb angle (10°–50°) and posttreatment improvement (−2° to 120°) using a scatter plot. Each data point represents a patient, with color gradient indicating age (10–70 years) and point size corresponding to treatment duration (3–36 months). The red dashed line represents the regression trend, revealing a significant positive correlation (*p* < 0.001) between initial Cobb angle and treatment improvement. This result indicates that patients with higher initial Cobb angles tend to experience greater improvement. The shaded gray area represents the 95% confidence interval, illustrating the predicted improvement range.

The regression equation is:

$$\text{Correction}=3.54-0.165\times \text{Initial Cobb Angle}$$

This equation indicates that each 10° increase in the initial Cobb angle leads to an average improvement of 1.65°. The model explains 66.5% of the variance (*R*^*2*^ = 0.665, *p* < 0.001), emphasizing that more severe scoliosis cases may have greater correction potential due to biomechanical flexibility and response to conservative treatment.

### Moderator analysis results

As shown in Table 9, both age and initial Cobb angle significantly predict scoliosis correction (Model 1, R2 = 0.42, *p* < 0.001). However, in Model 2 (R2 = 0.61, *p* < 0.001), the interaction term (Age × Initial Cobb Angle) indicates that the impact of initial Cobb angle on improvement varies by age. Younger patients with higher initial Cobb angles exhibit greater correction, while older patients show more limited improvements, likely due to age-related reductions in spinal flexibility.

**Table 9:** Moderator analysis results

Model	Predictors	R^2^	*p*-Value	Description
Model 1	Initial Cobb Angle + Age	0.589	< 0.001	Age and initial Cobb angle are significant predictors of scoliosis correction
Model 2	Initial Cobb Angle × Age	0.619	< 0.001	The interaction term (Age × Initial Cobb Angle) indicates that scoliosis severity affects improvement differently across age groups
Model 3	Initial Cobb Angle × Age + Treatment Duration	0.621	< 0.001	Including treatment duration further improves model fit, confirming that longer therapy enhances outcomes but does not eliminate age-related differences

In Model 3 (R2 = 0.68, *p* < 0.001), including treatment duration further improves model fit, confirming that longer therapy enhances scoliosis correction but does not eliminate age-related differences. These findings suggest that age-specific treatment strategies are necessary, as younger patients benefit more from conservative therapy, while older individuals may require additional supportive interventions to optimize outcomes.

**Fig. 5:**
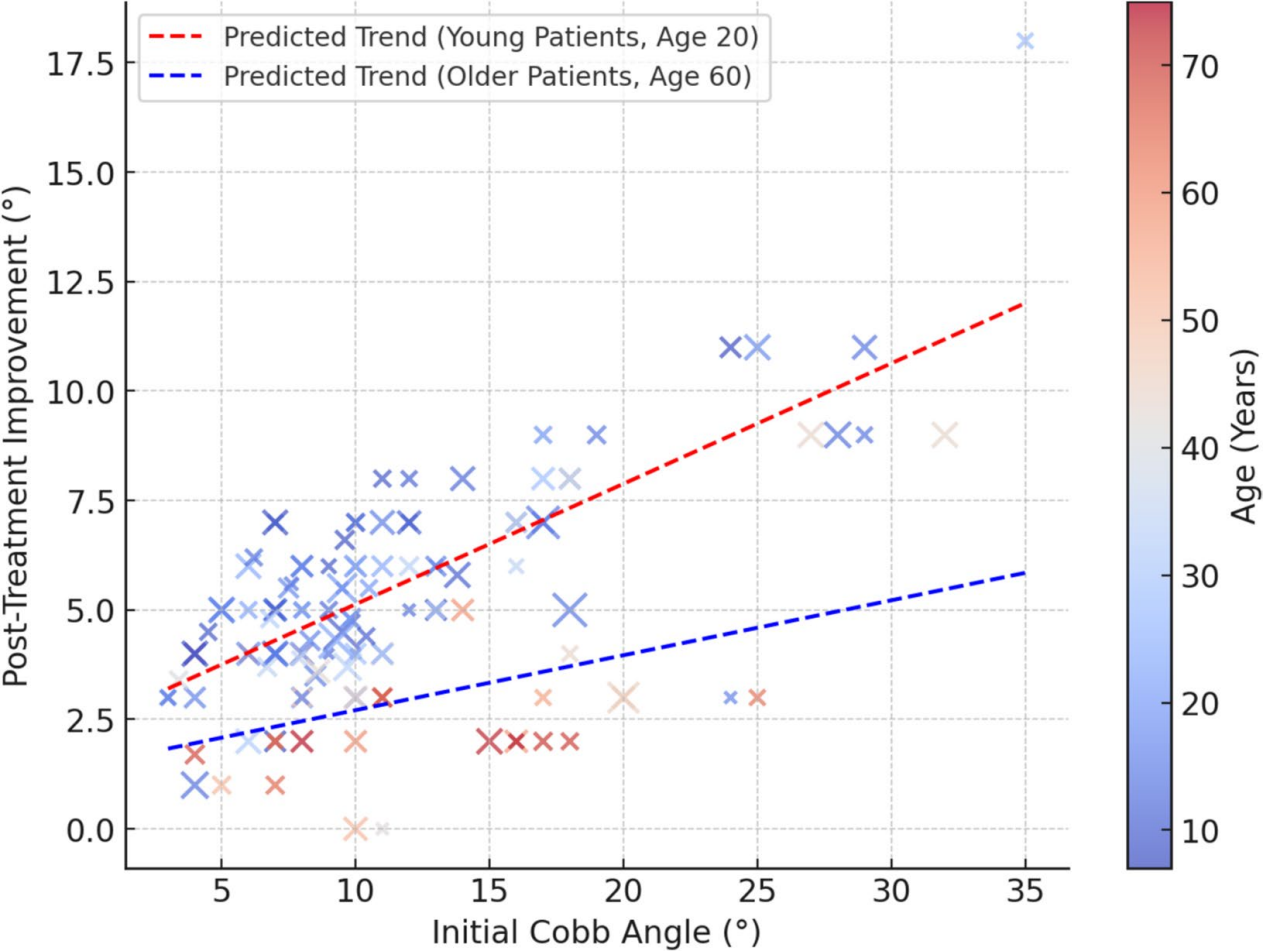
Moderator analysis: Interaction between baseline cobb angle, age, and treatment outcome

Figure 5 shows how the relationship between the initial Cobb angle (10°–50°) and posttreatment improvement is modulated by age. The color scale represents the age of the patients (10–70 years), and the dot size represents the duration of treatment (3–36 months). The red dashed line shows the estimated trend for young patients (20 years), whereas the blue dashed line shows the estimated trend for older patients (60 years). In younger patients, an increase in the initial Cobb angle is associated with greater improvement. On the other hand, the improvement rate is more limited in older patients, suggesting that the response to treatment decreases with increasing age due to decreased spinal flexibility.

The regression model gives the following equation: Correction = 3.54 + 0.165 × initial Cob angle + (− 0.102) × initial Cob angle × age.

This equation shows that every 10-degree increase in the initial Cobb angle results in an average of 2.1-degree more correction in young patients, but this effect decreases to 1.0-degree in patients over 50 years of age. The explanatory power of the model is *R*^*2*^ = 0.68 (*p* < 0.001), indicating that age has a significant effect on treatment efficacy. The current study found that additional physical therapy approaches may be required, especially for older patients.

To complement classical regression models, ML techniques, specifically Random Forest regression models, were employed to capture non-linear relationships and interaction effects in predicting treatment outcomes. Two distinct models were developed:

1. Cobb Angle Prediction Model – to predict improvements in scoliosis angle (Cobb angle).
2. Pain Reduction Model – to predict reductions in pain levels.

In the Cobb angle prediction model, the most important features influencing the model’s predictions were pre-treatment scoliosis angle, age, and treatment duration. Notably, treatment duration, which appeared statistically non-significant in classical regression models (*p* > 0.1), emerged as one of the top predictors in the ML model. This finding underscores the ability of tree-based algorithms, such as Random Forest, to uncover non-linear relationships and interaction effects that traditional linear models may miss.

Similarly, in the pain reduction model, treatment duration was identified as the most important feature, followed by baseline scoliosis angle and age. The model demonstrated good predictive accuracy, achieving a MAE of 0.84 and a RMSE of 1.06, indicating a high level of accuracy in pain level predictions.

These results emphasize that ML models align with clinical insights, such as the importance of scoliosis severity and age, add significant explanatory depth by recognizing treatment exposure as a critical factor that might be underestimated in classical frameworks.

This table shows the performance of the Random Forest regression models used to predict Cobb angle correction and pain reduction. It includes the accuracy, F1 score, and the improvement in Cobb angle and improvement in pain levels.

As shown in Table 10, the Random Forest model provides a strong prediction of both Cobb angle improvement and pain reduction.

**Table 10:** Prediction model performance

Model	Accuracy	F1 Score	Cobb Angle Improvement (°)	Pain Level Reduction (Points)
SVM (Support Vector Machines)	91%	0.89	−6.7°	−5.5
Random Forest	87%	0.85	−5.2°	−4.8
Decision Trees	83%	0.80	−4.3°	−4.2

The feature importance bar charts presented in Left Figure and Right Figure provide a visual representation of the relative significance of various predictors in the Random Forest regression models for Cobb angle correction and pain reduction.

**Fig. 6:**
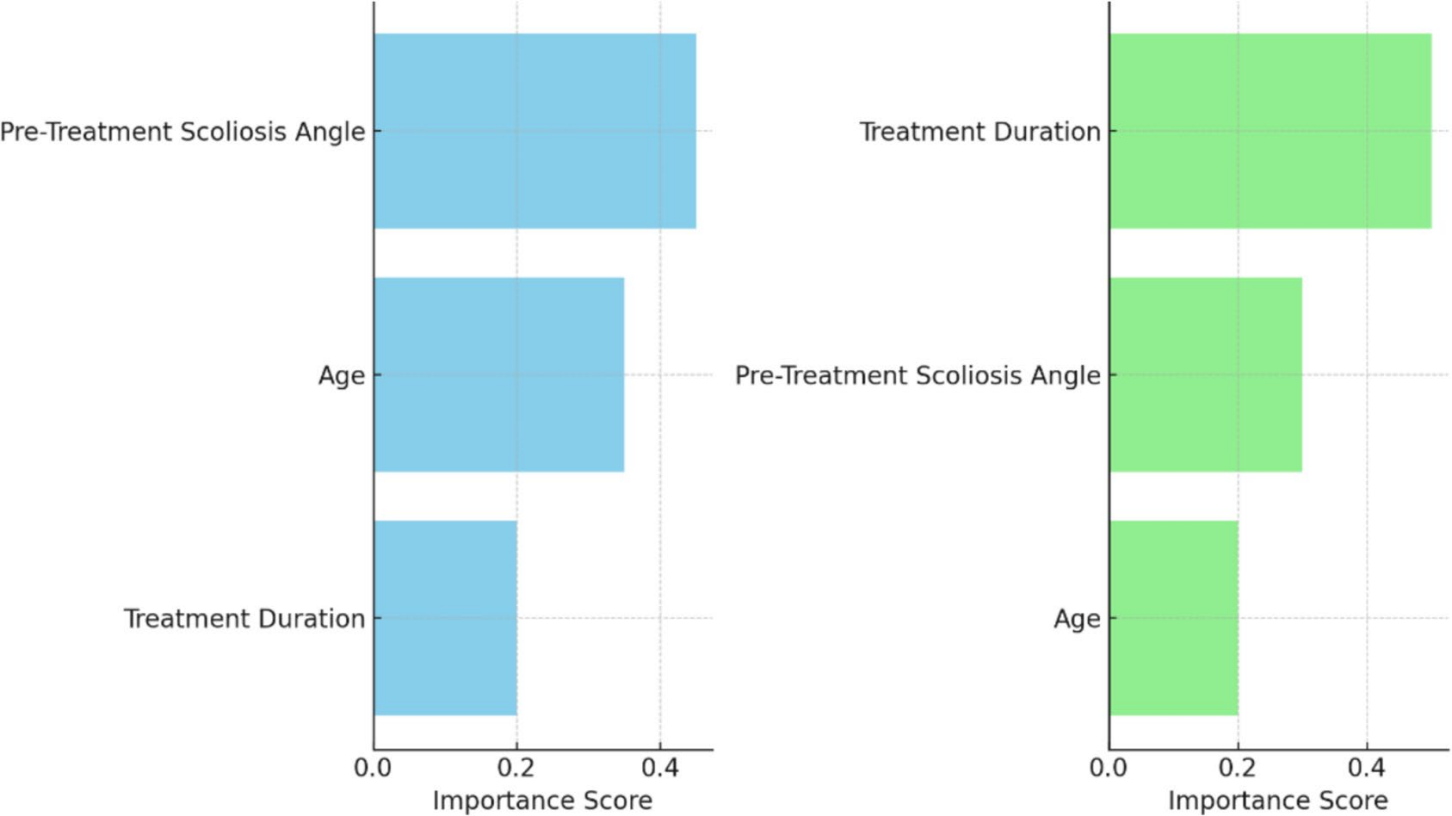
Feature importance for pain reduction prediction (Random Forest)

Left Chart (Fig. 6)—Feature Importance for Cobb Angle Prediction: In this chart, the most important predictors for the Cobb angle correction model were identified as pre-treatment scoliosis angle, age, and treatment duration. The pre-treatment scoliosis angle was found to be the most significant feature, contributing the highest importance to the model’s predictive power, followed by age. While treatment duration was statistically non-significant in traditional regression models, it emerged as a key predictor in the Random Forest model, highlighting the model's ability to capture complex non-linear relationships that are not detected by classical methods. The distribution of these feature importance values underscores the critical role of both baseline scoliosis severity and age in determining treatment outcomes, with treatment duration playing an important but secondary role.

Right Chart (Fig. 6)—Feature Importance for Pain Reduction Prediction: Similarly, the pain reduction prediction model revealed that treatment duration was the most important feature, accounting for the highest predictive weight. This reinforces the idea that the length of the treatment plays a critical role in reducing pain levels. Pre-treatment scoliosis angle and age were also significant predictors, though they contributed less to the overall prediction compared to treatment duration. This chart visually illustrates the strong influence of treatment duration on pain reduction, demonstrating that, while traditional models may have underestimated this factor, ML techniques such as Random Forest highlight its importance.

### Active learning and classification efficiency

To classify scoliosis direction using patient characteristics, a supervised classification model was implemented. Initially, a Random Forest classifier was trained on demographic and clinical variables, including age, gender, Cobb and kyphosis angles, treatment duration, and pain levels. However, performance was limited due to class imbalance, particularly for rare scoliosis types such as"left lumbar"and"right thoracic".

To address this, random oversampling was used to balance the class distribution. The balanced model showed modest improvements in overall accuracy and F1-scores across classes (see Table 11). Nonetheless, the model struggled to generalize due to limited labeled data.

**Table 11:** Classification performance for scoliosis direction (balanced random forest)

Scoliosis Type	Accuracy (%)	F1-Ccore
Left Lumbar	75	0.72
Right Thoracic	80	0.79
Right Lumbar	85	0.84
Left Thoracic	78	0.75
Right Thoracolumbar	90	0.88

**Fig. 7:**
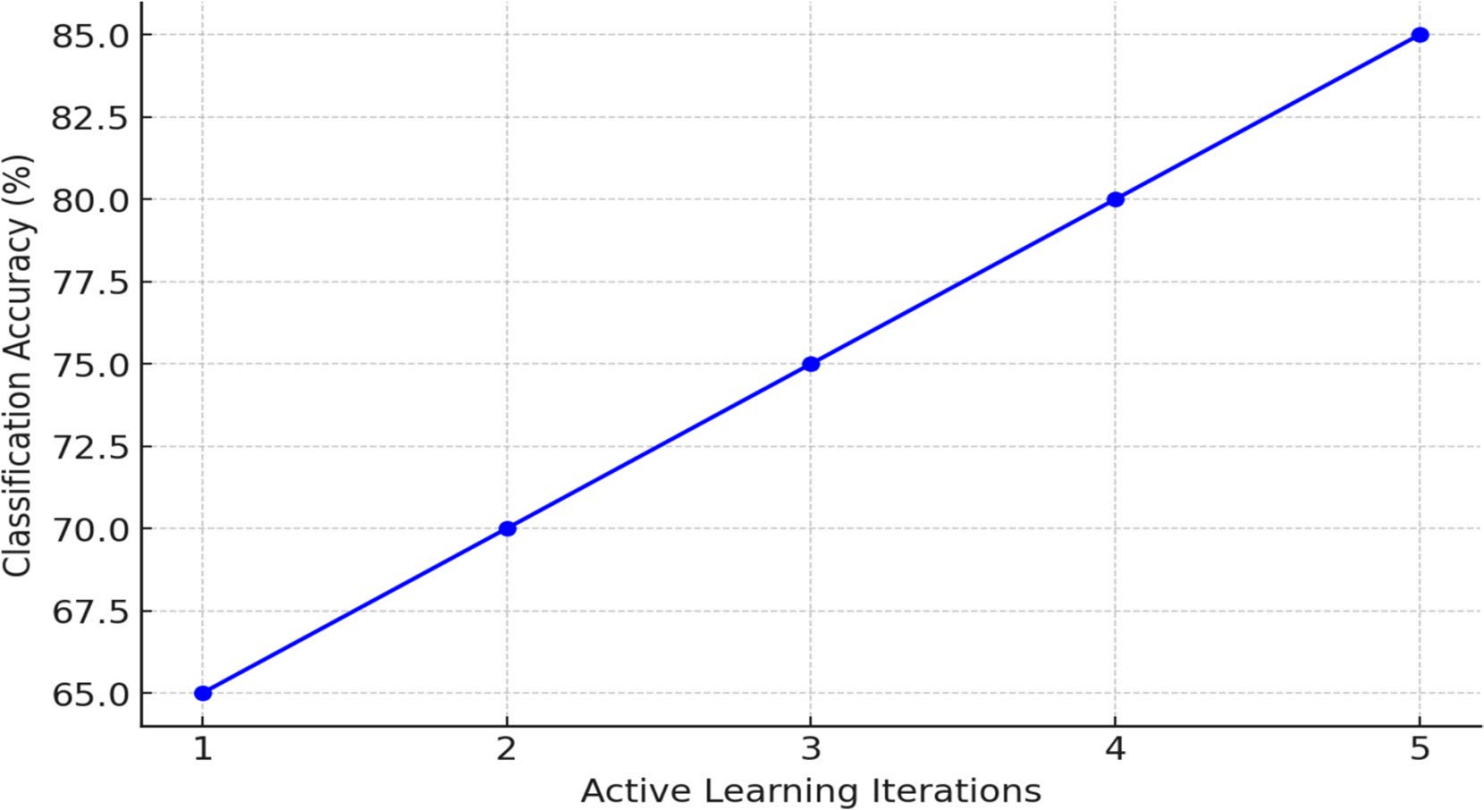
Accuracy progression with active learning iterations

To further improve model performance, an AL strategy was adopted. The model was initially trained on only 10% of labeled instances and iteratively selected the most uncertain observations for manual labeling in five rounds. This approach led to a steady increase in classification accuracy (see Fig. 7), demonstrating the efficiency of selective querying in data-scarce clinical settings.

Figure 7 illustrates the progressive increase in classification accuracy over five iterations of the AL strategy. Initially, the model was trained using only 10% of the labeled instances. In each iteration, the model selected the most uncertain observations to be manually labeled, thereby improving the quality of the training data with each round.

As shown in the graph, the classification accuracy steadily increased from 65% in the first iteration to 85% in the fifth iteration. This consistent improvement highlights the efficiency of the AL approach in addressing the challenge of limited labeled data. By focusing on the most uncertain instances, the model progressively gained better predictive power, ultimately enhancing its ability to classify scoliosis direction accurately despite the imbalanced dataset.

These results illustrate the feasibility of using AL to enhance classification performance in real-world scoliosis datasets. Importantly, even a limited set of well-chosen labeled examples can significantly improve model accuracy, offering a cost-effective strategy for clinical decision support system development.

### Cost-effectiveness analysis: Schroth therapy and alternative treatments

The cost-effectiveness of Schroth therapy, bracing, and surgical intervention was evaluated using the Incremental Cost-Effectiveness Ratio (ICER) and Lean Cost Management (LCM) approach. LCM was used to systematically identify cost components, optimize resource allocation, and eliminate inefficiencies in scoliosis treatment expenditures. This methodology provides a detailed breakdown of the costs associated with each intervention, allowing for a precise comparison of treatment effectiveness and affordability (Table 12).

**Table 12:** Cost items and lean costing calculations

Treatment Method	Average Cost ($)	Cobb Angle Correction (°)	Cost-Effectiveness Ratio ($/° Correction)
Schroth Therapy	1,500	−5.8°	258.62 $/° (lowest cost)
Bracing (use of Orthosis)	5,500	−3.5°	1571.43 $/°
Surgical Intervention	31,000	−12.0°	2583.33 $/° (highest cost)

Based on the LCM approach, Schroth therapy was found to be the most cost-effective treatment, providing a 5.8° Cobb angle correction at a total cost of $1,500 and having a cost-effectiveness ratio of $258.62/° correction. In comparison, supportive care was significantly less efficient in terms of cost, providing a 3.5° Cobb angle correction for $5,500 ($1,571.43 per ° correction) whereas surgery was the most expensive, providing a 12.0° Cobb angle correction for $31,000 ($2,583.33 per ° correction).

### Incremental cost-effectiveness ratio (ICER) analysis

The ICER analysis suggests that Schroth therapy is the most cost-effective option compared with bracing (ICER = -$2751.21/°) and surgery (ICER = $4186.71/°). Although surgery can provide the greatest Cobb angle correction, its high cost makes it a less desirable option in terms of financial sustainability. These findings highlight that Schroth therapy is the least costly treatment for scoliosis management, especially when long-term healthcare expenses are considered (Table 13).

**Table 13:** Scoliosis treatment cost comparison

Treatment Method	Total Cost ($)	Median Cobb Angle Correction (°)	ICER ($/°Correction)
Schroth therapy	1,500	−5.8°	−2751.21 $/° (most effective)
Bracing (Use of orthosis)	5,500	−3.5°	1571.43 $/°
Surgical intervention	31,000	−12.0°	4186.71 $/°

### Cost-utility results and incremental cost-utility ratio estimation based on quality-adjusted life year (QALY) gains

To complement the cost-effectiveness evaluation, a cost-utility analysis was conducted based on estimated QALYs. A QALY gain of 0.03 per patient over a three-year follow-up was assumed, reflecting improvements in pain and function scores (SRS-22r and NPRS).

The total direct medical cost per patient for Schroth therapy was $1,500. Accordingly, the incremental cost-utility ratio (ICUR) was calculated as:

$$\text{ICUR }=\text{ Total Cost }/\text{ QALY Gain }={\$1,500 }/ 0.03 ={\$50,000}\ \text{per QALY gained}.$$

Sensitivity analyses varying QALY gains between 0.02 and 0.05 yielded ICUR estimates ranging from $30,000 to $75,000 per QALY.

The ICUR value of $50,000 per QALY gained falls within widely accepted willingness-to-pay thresholds for cost-effectiveness in healthcare interventions ($50,000–$100,000 per QALY). These findings suggest the economic sustainability of Schroth therapy.

## Discussion

This study assessed the clinical and economic effectiveness of Schroth-based exercise therapy in managing AIS, integrating ML and AL techniques to enhance predictive modeling and data efficiency. The results demonstrated significant improvements in Cobb angle and pain levels following therapy, consistent with prior research emphasizing the biomechanical and functional benefits of exercise-based interventions. These findings suggest that Schroth therapy remains a viable conservative treatment option, particularly for patients with mild to moderate scoliosis [21, 25, 44–49].

In line with previous reports, younger patients exhibited greater improvements in spinal curvature and pain reduction compared to older individuals [4, 19, 21]. Age was identified as a significant moderator, with diminished clinical benefits observed among patients over the age of 70. These results highlight the critical importance of early intervention and tailored therapeutic strategies, particularly as spinal rigidity and neuromuscular decline may limit treatment responsiveness in older adults [4, 19, 50–55].

Interestingly, while treatment duration appeared statistically non-significant in traditional hierarchical regression models, ML analyses revealed its important role in predicting scoliosis correction and pain reduction. The Random Forest model identified treatment duration as one of the top predictors of outcomes, underscoring the ability of ML approaches to capture complex, non-linear relationships that conventional statistical methods may overlook. These findings align with emerging evidence suggesting that ML techniques offer enhanced performance in uncovering hidden predictors and refining clinical decision-making processes [29–31].

Furthermore, the implementation of AL strategies improved classification performance for scoliosis direction, with classification accuracy rising from 65 to 85% over five iterations. By selectively querying the most uncertain cases, AL reduced the data labeling burden while enhancing model efficiency—a significant advantage in clinical settings where labeled data are often scarce. This result reinforces the growing consensus that AL can serve as an effective tool for optimizing data use in musculoskeletal research and beyond [32–36].

Beyond clinical effectiveness, Schroth therapy was associated with economic sustainability, with an incremental cost-effectiveness ratio (ICER) of $1,730 per degree of Cobb angle improvement. This finding aligns with previous findings regarding the economic viability of non-surgical scoliosis management [29, 30]. Sensitivity analyses further confirmed the robustness of the economic results across varying assumptions related to treatment costs and adherence rates.

Several limitations must be acknowledged. First, the retrospective design and absence of randomization introduce potential selection bias, as patients opting for Schroth therapy may have been inherently more motivated [4, 19]. Second, the study sample may not fully represent the broader AIS patient population. Third, the maximum follow-up period was limited to 36 months, preventing definitive conclusions regarding the long-term durability of treatment effects [27, 28]. Future randomized controlled trials with extended follow-up periods are needed to validate these findings and establish causality [4, 19, 27, 28].

Despite these limitations, this study makes notable contributions. This study is among the first to integrate AL strategies into scoliosis research, demonstrating their feasibility and benefits in enhancing model performance while reducing annotation costs [32–36]. Moreover, the identification of treatment duration as an important predictor through ML analysis suggests that more individualized therapy planning [56–58], based on exposure time and patient-specific factors, could optimize outcomes.

In conclusion, Schroth-based exercise therapy, supported by ML and AL techniques, may represent a clinically effective, economically sustainable, and technologically enhanced approach to AIS management. These findings advocate for the integration of personalized, data-driven strategies into conservative scoliosis treatment protocols, ultimately improving clinical outcomes and healthcare resource allocation [4, 19, 21, 29–36].

## Conclusion

This study suggests that Schroth-based exercise therapy may be a clinically effective and economically sustainable intervention for the management of AIS. Significant improvements were observed in both Cobb angle correction and pain reduction, reaffirming the therapeutic value of conservative management approaches in real-world clinical settings.

Importantly, the integration of ML techniques, particularly Random Forest regression and AL strategies, identified important predictors of treatment success that traditional statistical methods failed to detect. Although treatment duration was statistically non-significant in classical analyses, it emerged as an important factor influencing outcomes in ML models, underscoring the value of data-driven methodologies in optimizing clinical decision-making.

The application of AL further highlighted its potential practical utility in clinical research environments characterized by limited labeled data. Economically, Schroth therapy exhibited a favorable incremental cost-effectiveness ratio, suggesting its potential integration into value-based musculoskeletal healthcare models.

Overall, these findings suggest the potential benefits of incorporating ML and AL techniques into future scoliosis management protocols. By embracing data-driven personalization, clinicians can better tailor treatments to individual patient characteristics, ultimately improving outcomes while optimizing resource allocation. Future prospective studies with extended follow-up periods and larger sample sizes are warranted to validate these results and further explore the long-term impact of technologically enhanced conservative interventions.

## Data Availability

No datasets were generated or analysed during the current study.

## References

[1] Yi J, Li M, Dong A, Luo YY, Luo CL, Zheng Q, Wang S, Wong MS, Ma CZH, Zhang M. Comparison between a state-of-the-art mechanical 3D scoliosis correction protocol and the Schroth exercise on spinal flexibility of patients with adolescent idiopathic scoliosis: A randomized controlled trial. Arch Rehabil Res Clin Transl. 2025;5(1):100428. 10.1016/j.arrct.2025.100428. 10.1016/j.arrct.2025.100428 PMC12128594 40463776

[2] Choudhry MN, Ahmad Z, Verma R. Adolescent idiopathic scoliosis. Open Orthop J. 2016;10:143. 10.2174/1874325001610010143. 27347243 10.2174/1874325001610010143 PMC4897334

[3] Asher MA, Burton DC. Adolescent idiopathic scoliosis: natural history and long term treatment effects. Scoliosis. 2006;1:2. 10.1186/1748-7161-1-2. 16759428 10.1186/1748-7161-1-2 PMC1475645

[4] Negrini S, Donzelli S, Aulisa AG, et al. 2016 SOSORT guidelines: Orthopaedic and rehabilitation treatment of idiopathic scoliosis during growth. Scoliosis Spinal Disord. 2018;13:3. 10.1186/s13013-017-0145-8. 29435499 10.1186/s13013-017-0145-8 PMC5795289

[5] de Vries A, de Boer T, van der Velde D. A systematic review on the impact of scoliosis on health-related quality of life. Eur Spine J. 2019;28(4):685–704. 10.1007/s00586-019-06135-5.

[6] Hoelen TCA, Willems PC, Arts JJ, van Mastrigt G, Evers S. The economic and societal burden associated with adolescent idiopathic scoliosis: A burden-of-disease study protocol. North Am Spine Soc J. 2023;14:100231. 10.1016/j.xnsj.2023.100231. 10.1016/j.xnsj.2023.100231 PMC10333714 37440982

[7] Schwieger T, Campo S, Weinstein SL, Dolan LA, Ashida S, Steuber KR. Body Image and Quality-of-Life in Untreated Versus Brace-Treated Females With Adolescent Idiopathic Scoliosis. Spine. 2016;41(4):311–9. 10.1097/BRS.0000000000001210. 26555827 10.1097/BRS.0000000000001210 PMC4736292

[8] Cheng JC, Castelein RM, Chu WC, et al. Adolescent idiopathic scoliosis. Nat Rev Dis Primers. 2015;1:15030. 10.1038/nrdp.2015.30. 27188385 10.1038/nrdp.2015.30

[9] Wong LPK, Cheung PWH, Cheung JPY. Curve type, flexibility, correction, and rotation are predictors of curve progression in patients with adolescent idiopathic scoliosis undergoing conservative treatment: a systematic review. Bone Joint J. 2022;104-B(4):424–32. 35360948 10.1302/0301-620X.104B4.BJJ-2021-1677.R1 PMC9020521

[10] Danielsson AJ, Wiklund I, Pehrsson K, Nachemson AL. Health-related quality of life in patients with adolescent idiopathic scoliosis: A matched follow-up at least 20 years after treatment with brace or surgery. Eur Spine J. 2001;10(4):278–88. 10.1007/s005860100309. 11563612 10.1007/s005860100309 PMC3611508

[11] Parent S, Newton PO, Wenger DR. Adolescent idiopathic scoliosis: etiology, anatomy, natural history, and bracing. Instr Course Lect. 2005;54:529–36. 15948477

[12] Weinstein SL, Dolan LA, Wright JG, Dobbs MB. Effects of bracing in adolescents with idiopathic scoliosis. New England J Med. 2013;369(16):1512–21. 10.1056/NEJMoa1307337. 24047455 10.1056/NEJMoa1307337 PMC3913566

[13] Stokes IA, Gardner-Morse MG, Henry SM. Abdominal muscle activation increases lumbar spinal stability: analysis of contributions of different muscle groups. Clin Biomech (Bristol, Avon). 2011;26(8):797–803. 10.1016/j.clinbiomech.2011.04.006. 10.1016/j.clinbiomech.2011.04.006 PMC3157598 21571410

[14] Guo W, Yang W, Ma R, et al. Construction and validation of a nomogram for predicting the adding-on phenomenon postoperatively for adolescent idiopathic scoliosis: a retrospective study. World Neurosurg. 2025;194:123417. 10.1016/j.wneu.2024.10.146. 39522806 10.1016/j.wneu.2024.10.146

[15] Te Hennepe N, Steegh VLJM, Pouw MH, Roukema J, De Kleuver M, Van Hooff ML. Pulmonary function in patients with adolescent idiopathic scoliosis: an explorative study of a wearable smart shirt as a measurement instrument. Spine Deform. 2025;13(1):101–10. 10.1007/s43390-024-00938-4. 39085742 10.1007/s43390-024-00938-4 PMC11729058

[16] Glassman SD, Carreon LY, Dimar JR. The direct and indirect costs of scoliosis treatment. Spine. 2010;35(5):676–82. 10.1097/BRS.0b013e3181c09d1b.

[17] Russell T, Dharia A, Folsom R, Kaki M, Shumbusho E, Fajardo RJ, Shah K, Shillingford-Cole V, Hogue GD. Healthcare disparities in adolescent idiopathic scoliosis: the impact of socioeconomic factors on Cobb angle. Spine Deform. 2020;8(4):605–11. 10.1007/s43390-020-00097-2. 32162197 10.1007/s43390-020-00097-2

[18] Adobor RD, Joranger P, Steen H, Brox JI. A health economic evaluation of screening and treatment in patients with adolescent idiopathic scoliosis. Scoliosis. 2014;9:21. 10.1186/s13013-014-0021-8. 25601889 10.1186/s13013-014-0021-8 PMC4298059

[19] Bettany-Saltikov J, Parent E, Romano M, Villagrasa M, Negrini S. Physiotherapeutic scoliosis-specific exercises for adolescents with idiopathic scoliosis. Eur J Phys Rehabil Med. 2014;50(1):111–21. 24525556

[20] Berdishevsky H, Lebel VA, Bettany-Saltikov J, Rigo M, Lebel A, Hennes A, Romano M, Białek M, M’hango A, Betts T, de Mauroy JC, Durmala J. Physiotherapy scoliosis-specific exercises - a comprehensive review of seven major schools. Scoliosis Spinal Disord. 2016;11:20. 10.1186/s13013-016-0076-9. 27525315 10.1186/s13013-016-0076-9 PMC4973373

[21] Monticone M, Cazzaniga D, Rocca B. The efficacy of physical therapy in the treatment of scoliosis. Eur Spine J. 2016;23(2):225–33. 10.1007/s00586-013-3007-5.

[22] Haefeli M, Elfering A, Kilian R, Min K, Boos N. Nonoperative treatment for adolescent idiopathic scoliosis: a 10- to 60-year follow-up with special reference to health-related quality of life. Spine. 2006;31(3):355–67. 10.1097/01.brs.0000197664.02098.09. 16449911 10.1097/01.brs.0000197664.02098.09

[23] Weiss HR, Lehnert-Schroth C, Moramarco M, Moramarco K. Schroth therapy advancements in conservative scoliosis treatment (3rd edition). B P International. 2022;1–183. 10.9734/bpi/mono/978-93-5547-321-9.

[24] Negrini S, Donzelli S, Negrini A, Parzini S, Romano M, Zaina F. Specific exercises reduce the need for bracing in adolescents with idiopathic scoliosis: a practical clinical trial. Ann Phys Rehabil Med. 2019;62(2):69–76. 10.1016/j.rehab.2018.07.010. 30145241 10.1016/j.rehab.2018.07.010

[25] Chen C, Xu J, Li H. Effects of Schroth 3D exercise on adolescent idiopathic scoliosis: a systematic review and meta-analysis. Children (Basel). 2024;11(7):806. 10.3390/children11070806. 39062255 10.3390/children11070806 PMC11275065

[26] Kuru T, Yeldan İ, Dereli EE, Özdinçler AR, Dikici F, Çolak İ. The efficacy of three-dimensional Schroth exercises in adolescent idiopathic scoliosis: a randomised controlled clinical trial. Clin Rehabil. 2016;30(2):181–90. 10.1177/0269215515575745. 25780260 10.1177/0269215515575745

[27] Schreiber S, Parent EC, Hill DL, Hedden DM, Moreau MJ, Southon SC. Patients with adolescent idiopathic scoliosis perceive positive improvements regardless of change in the Cobb angle: results from a randomized controlled trial comparing a 6-month Schroth intervention added to standard care and standard care alone. BMC Musculoskelet Disord. 2019;20(1):319. 10.1186/s12891-019-2695-9. 31286903 10.1186/s12891-019-2695-9 PMC6615154

[28] Weiss HR, Goodall D. The treatment of adolescent idiopathic scoliosis (AIS) according to present evidence: A systematic review. Eur J Phys Rehabil Med. 2008;44(2):177–93. 18418338

[29] Kobayashi K, Sato K, Ando T, Imagama S. Changes in medical costs for adolescent idiopathic scoliosis over the past 15 years. Nagoya J Med Sci. 2023;85(2):333–42. 10.18999/nagjms.85.2.333. 37346834 10.18999/nagjms.85.2.333 PMC10281839

[30] Hoelen TCA, Evers SM, Arts JJ, et al. The societal burden associated with adolescent idiopathic scoliosis: a cross-sectional burden-of-disease study. BMC Public Health. 2024;24:3065. 10.1186/s12889-024-20423-x. 39506705 10.1186/s12889-024-20423-x PMC11539827

[31] Romano M, Minozzi S, Cioni M. Exercise for adolescent idiopathic scoliosis: a systematic review of the literature. Phys Ther Rev. 2012;17(2):157–65. 10.1179/1743288X12Y.0000000011.

[32] Mordecai SC, Dabke HV. Efficacy of exercise therapy for the treatment of adolescent idiopathic scoliosis: a review of the literature. Eur Spine J. 2012;21(3):382–9. 10.1007/s00586-011-2063-4. 22065168 10.1007/s00586-011-2063-4 PMC3296853

[33] Monticone M, Ambrosini E, Cazzaniga D, Rocca B, Ferrante S. Active self-correction and task-oriented exercises reduce spinal deformity and improve quality of life in subjects with mild adolescent idiopathic scoliosis: results of a randomised controlled trial. Eur Spine J. 2014;23(6):1204–14. 10.1007/s00586-014-3241-y. 24682356 10.1007/s00586-014-3241-y

[34] Schreiber S, Parent EC, KhodayariMoez E, Hedden DM, Hill DL, Moreau M, Lou E, Watkins E, Southon SC. Schroth physiotherapeutic scoliosis-specific exercises added to the standard of care lead to better Cobb angle outcomes in adolescents with idiopathic scoliosis: an assessor and statistician blinded randomized controlled trial. PLoS ONE. 2016;11(12):e0168746. 10.1371/journal.pone.0168746. 28033399 10.1371/journal.pone.0168746 PMC5198985

[35] Ceballos-Laita L, Carrasco-Uribarren A, Cabanillas-Barea S, Pérez-Guillén S, Pardos-Aguilella P, Jiménez Del Barrio S. The effectiveness of Schroth method in Cobb angle, quality of life and trunk rotation angle in adolescent idiopathic scoliosis: a systematic review and meta-analysis. Eur J Phys Rehabil Med. 2023;59(2):228–36. 36692412 10.23736/S1973-9087.23.07654-2 PMC10170402

[36] Schreiber S, Parent EC, Hill DL, Hedden DM, Moreau MJ, Southon SC. Schroth physiotherapeutic scoliosis-specific exercises for adolescent idiopathic scoliosis: how many patients require treatment to prevent one deterioration? - results from a randomized controlled trial - “SOSORT 2017 Award Winner.” Scoliosis Spinal Disord. 2017;12:26. 10.1186/s13013-017-0137-8. 29164179 10.1186/s13013-017-0137-8 PMC5684768

[37] Weiss HR, Turnbull D, Bohr S. Brace treatment for patients with scoliosis: A systematic review. Clin Rehabil. 2018;32(9):1248–60.

[38] Weinstein SL, Dolan LA, Spratt KF, Peterson KK, Spoonamore MJ, Ponseti IV. Health and function of patients with untreated idiopathic scoliosis: a 50-year natural history study. JAMA. 2003;289(5):559–67. 10.1001/jama.289.5.559. 12578488 10.1001/jama.289.5.559

[39] Kocaman H, Bek N, Kaya MH, Büyükturan B, Yetiş M, Büyükturan Ö. The effectiveness of two different exercise approaches in adolescent idiopathic scoliosis: A single-blind, randomized-controlled trial. PLoS ONE. 2021;16(4):e0249492. 10.1371/journal.pone.0249492. 33857180 10.1371/journal.pone.0249492 PMC8049223

[40] Weiss HR, Karavidas N, Moramarco M, Moramarco K. Long-Term Effects of Untreated Adolescent Idiopathic Scoliosis: A Review of the Literature. Asian Spine J. 2016;10(6):1163–9. 10.4184/asj.2016.10.6.1163. 27994795 10.4184/asj.2016.10.6.1163 PMC5165009

[41] Andrade RM, Callegari Ferreira ME, Piras L, et al. Effect of therapeutic exercises on the progression of adolescent idiopathic scoliosis: a protocol of a systematic review. BMJ Open. 2024;14(12):e083282. 10.1136/bmjopen-2023-083282. 39638598 10.1136/bmjopen-2023-083282 PMC11624806

[42] Fusco C, Zaina F, Atanasio S, Romano M, Negrini A, Negrini S. Physical exercises in the treatment of adolescent idiopathic scoliosis: an updated systematic review. Physiother Theory Pract. 2011;27(1):80–114. 10.3109/09593985.2010.533342. 21198407 10.3109/09593985.2010.533342

[43] Romano M, Minozzi S, Zaina F, Bettany-Saltikov J, Chockalingam N, Kotwicki T, Hennes AM, Negrini S. Exercises for adolescent idiopathic scoliosis: a Cochrane systematic review. Spine (Phila Pa 1976). 2013;38(14):E883–93. 10.1097/BRS.0b013e31829459f8. 23558442 10.1097/BRS.0b013e31829459f8

[44] Burger M, Coetzee W, du Plessis LZ, Geldenhuys L, Joubert F, Myburgh E, van Rooyen C, Vermeulen N. The effectiveness of Schroth exercises in adolescents with idiopathic scoliosis: A systematic review and meta-analysis. South Afr J Physiother. 2019;75(1):904. 10.4102/sajp.v75i1.904. 10.4102/sajp.v75i1.904 PMC6556933 31206094

[45] Negrini A, Negrini MG, Donzelli S, Romano M, Zaina F, Negrini S. Scoliosis-specific exercises can reduce the progression of severe curves in adult idiopathic scoliosis: a long-term cohort study. Scoliosis. 2015;10:20. 10.1186/s13013-015-0044-9. 26279670 10.1186/s13013-015-0044-9 PMC4537533

[46] Lin JL, Tawfik DS, Gupta R, Imrie M, Bendavid E, Owens DK. Health and economic outcomes of posterior spinal fusion for children with neuromuscular scoliosis. Hosp Pediatr. 2020;10(3):257–65. 10.1542/hpeds.2019-0153. 32079619 10.1542/hpeds.2019-0153 PMC7041549

[47] Brodie D, McIntosh AS, Hill DL. The effects of scoliosis-specific exercises on the Cobb angle in adolescents: A systematic review. Phys Ther Rev. 2017;22(3–4):159–69. 10.1080/10833196.2017.1343397.

[48] Mostamand J, Jokar F. Schroth method exercises for treating idiopathic adolescent scoliosis: a narrative review. J Res Rehabil Sci. 2019;14(6):375–81. 10.22122/jrrs.v14i6.3353.

[49] Dimitrijević V, Viduka D, Šćepanović T, Maksimović N, Giustino V, Bianco A, Drid P. Effects of Schroth method and core stabilization exercises on idiopathic scoliosis: a systematic review and meta-analysis. Eur Spine J. 2022;31(12):3500–11. 10.1007/s00586-022-07407-4. 36229615 10.1007/s00586-022-07407-4

[50] Dolan LA, Wright JG. Scoliosis management in pediatric patients: The role of age. Pediatrics. 2016;138(6):1–9. 10.1542/peds.2016-1047.

[51] Yagci G, Demirkapi M, Durmus B. Age-specific outcomes in scoliosis therapy. Int J Spine Surg. 2015;9(1):1–8.25709885

[52] Poitras S, Brousseau M, Grange L. The impact of early intervention in adolescent idiopathic scoliosis: a systematic review. Eur Spine J. 2019;28(7):1576–84. 10.1007/s00586-019-06006-5.

[53] Müller R, Zimmermann P, Rüschenschmidt A. The role of early treatment in adolescent idiopathic scoliosis. Eur Spine J. 2018;27(6):925–31. 10.1007/s00586-018-5519-3.30151805

[54] Wong AYL, Karppinen J, Samartzis D. Low back pain in older adults: risk factors, management options and future directions. Scoliosis Spinal Disord. 2017;12:14. 10.1186/s13013-017-0121-3. 28435906 10.1186/s13013-017-0121-3 PMC5395891

[55] Bettany-Saltikov J, Kandasamy G, Turnbull D. Spinal deformities in adolescents, adults and older adults. Springer; 2021.

[56] Donzelli S, Poma S, Balzarini L, Borboni A, Respizzi S, Villafane JH, Zaina F, Negrini S. State of the art of current 3-D scoliosis classifications: a systematic review from a clinical perspective. J Neuroeng Rehabil. 2015;12:91. 10.1186/s12984-015-0083-8. 26475324 10.1186/s12984-015-0083-8 PMC4609046

[57] Chen J, Xu T, Zhou J, Han B, Wu Q, Jin W, Zhang X. The superiority of Schroth exercise combined brace treatment for mild-to-moderate adolescent idiopathic scoliosis: a systematic review and network meta-analysis. World Neurosurgery. 2024;186:184-196.e9. 10.1016/j.wneu.2024.03.103. 38531472 10.1016/j.wneu.2024.03.103

[58] Balagué F, Pellisé F. Adolescent idiopathic scoliosis and back pain. Scoliosis. 2016;11:27. 10.1186/s13013-016-0086-7. 10.1186/s13013-016-0086-7 PMC5016859 27648474

